# Long-term safety of intravenous human umbilical cord blood-derived mesenchymal stem cell therapy in rheumatoid arthritis: a 5-year follow-up study

**DOI:** 10.1093/stcltm/szag062

**Published:** 2026-08-05

**Authors:** Eun Hye Park, Min Jung Kim, Kyung-Sun Kang, Kichul Shin

**Affiliations:** Department of Internal Medicine, Division of Rheumatology, Chung-Ang University College of Medicine, Seoul 06974, Republic of Korea; Department of Internal Medicine, Division of Rheumatology, Chung-Ang University Gwangmyeong Hospital, Gwangmyeong 14353, Republic of Korea; Department of Internal Medicine, Division of Rheumatology, Seoul Metropolitan Government–Seoul National University Boramae Medical Center, Seoul 07061, Republic of Korea; Institute for Stem Cell Regenerative Medicine, Kangstem Biotech, Seoul 06181, Republic of Korea; Adult Stem Cell Research Center, College of Veterinary Medicine, Seoul National University, Seoul 08826, Republic of Korea; Department of Internal Medicine, Division of Rheumatology, Seoul Metropolitan Government–Seoul National University Boramae Medical Center, Seoul 07061, Republic of Korea; Department of Internal Medicine, College of Medicine, Seoul National University, Seoul 03080, Republic of Korea

**Keywords:** rheumatoid arthritis, mesenchymal stem cells, safety

## Abstract

**Background/purpose:**

Mesenchymal stem cell (MSC) therapy offers promise for treating autoimmune diseases due to its strong immunomodulatory effects. We investigated the long-term safety of a single intravenous injection with human umbilical cord blood-derived (hUCB)-MSCs in patients with rheumatoid arthritis (RA).

**Methods:**

Patients with RA who met the 2010 ACR/EULAR classification criteria and received a single intravenous infusion of hUCB-MSCs (2.5 × 10^7^, 5.0 × 10^7^, 1.0 × 10^8^ cells) in a phase I trial (NCT02221258) entered this 5-year observational pilot study. Safety assessments were performed at 3, 6, and 12 months after infusion and annually thereafter. Safety endpoints included overall adverse events (AEs), serious adverse events (SAEs), and AEs of special interest.

**Results:**

Nine patients were treated. The most common AEs were osteoarthritis (44.4%) and nasopharyngitis (44.4%). SAEs occurred in 5 patients (55.6%); a serious infection (cellulitis) occurred in 1 patient in the 1.0 × 10^8^ group and resolved after treatment. Benign ovarian and breast tumors were reported in 2 patients 3 years post-infusion. No deaths, thromboembolism, or malignancies occurred during the follow-up period. Laboratory findings remained stable except for 1 case each of transient hypertriglyceridemia and mild eosinophilia. While DAS28 improved markedly by 3-6 months, disease activity gradually increased over 5 years, suggesting waning efficacy after a single infusion.

**Conclusion:**

The long-term safety profile of a single dose of intravenous hUCB-MSC in patients with RA appears acceptable; however, repeated-dose regimens may be needed for sustained disease control and further safety evaluation.

## Graphical abstract

**Graphical Abstract:**
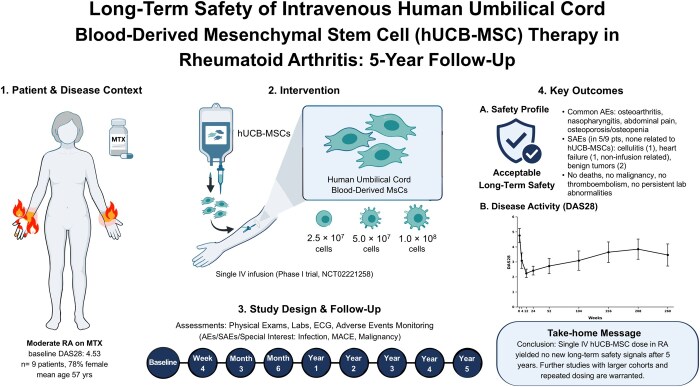

Significance statement This 5-year prospective follow-up provides long-term safety data for intravenous human umbilical cord blood-derived mesenchymal stem cell (hUCB-MSC) therapy for rheumatoid arthritis, for which long-term follow-up data remain limited. The absence of major safety signals, including serious infections, malignancies, and cardiovascular events, supports the clinical feasibility and tolerability of hUCB-MSCs, while further evaluation in larger, controlled studies is warranted.

## Introduction

Rheumatoid arthritis (RA) is a chronic systemic inflammatory disease characterized by synovial joint inflammation, which can lead to joint destruction and disability.^1^ During the past 2 decades, the introduction of biological disease-modifying anti-rheumatic drugs (bDMARDs) and targeted synthetic DMARDs (tsDMARDs) has significantly improved clinical outcomes in RA.^2^^,^^3^ However, the use of bDMARDs and tsDMARDs has been associated with an increased risk of infections.^4^^,^^5^ Notably, tumor necrosis factor-alpha (TNF-α) inhibitors are known to raise the risk of tuberculosis,^6^ rituximab is linked to hepatitis B virus,^7^ and Janus kinase (JAK) inhibitors have been associated with higher rates of herpes zoster.^8^ Additionally, concerns have been raised about a potentially increased overall risk of malignancy with JAK inhibitors compared to TNF inhibitors,^9^ as well as a higher risk of nonmelanoma skin cancer with TNF inhibitors.^10^

Given the importance of safety in choosing treatment for RA and the known adverse effects linked with bDMARDs and tsDMARDs, mesenchymal stem cell (MSC)-based therapies have become promising alternatives. MSCs are non-hematopoietic pluripotent cells capable of self-renewal, tissue regeneration, and immunomodulation.^8^ MSCs exert immunomodulatory effects by inhibiting dendritic cell maturation and natural killer cell activation, suppressing the proliferation and differentiation of T and B cells, promoting anti-inflammatory M2 macrophage polarization, and enhancing the induction of regulatory T (Treg) cells.^11^ Owing to these properties, the therapeutic potential of MSCs in RA has been studied extensively in preclinical^12^ and clinical studies.^13–16^

The main sources of human MSCs include various tissues, such as bone marrow (BM),^14^ adipose tissue,^13^ dental tissue,^17^ synovium,^18^ periosteum,^19^ umbilical cord,^16^ and umbilical cord blood.^15^ In patients with refractory RA, a phase Ib/IIa clinical trial demonstrated that intravenous infusion of allogeneic adipose-derived MSCs had a favorable overall safety profile.^13^ Similarly, intravenous administration of autologous BM-derived MSCs was associated with improvements in RA disease activity.^14^ Human umbilical cord blood-derived MSCs (hUCB-MSCs) offer distinct advantages over BM- and adipose-derived MSCs, including noninvasive collection, greater proliferative potential, and lower immunogenicity.^20^^,^^21^ Previously, we conducted a phase 1a trial of a single intravenous infusion of hUCB-MSCs in RA patients with moderate disease activity despite methotrexate (MTX) treatment. This study demonstrated an acceptable safety profile over a 4-week follow-up period.^15^ However, data on the long-term safety of MSC-based cellular therapies in RA remain limited. Therefore, in this 5-year prospective, observational exploratory pilot study, we aimed to investigate the long-term safety of a single intravenous infusion of hUCB-MSCs in patients with RA.

## Methods and materials

### Study design and population

This was a 5-year prospective observational study following participants from the CURE-iv trial (Clinical and safety assessment of hUCB-MSCs therapy for RhEumatoid arthritis patients administered intravenously; NCT02221258), a phase I, open-label, dose-escalation study.^15^ Participants were eligible if they were 18 years or older, met the 2010 American College of Rheumatology (ACR)/European League Against Rheumatism classification criteria for RA,^22^ and had a baseline disease activity score 28 (DAS28) greater than 3.2 despite MTX treatment.^15^

In the CURE-iv trial, patients with RA received a single intravenous infusion of 2.5 × 10^7^, 5 × 10^7^, or 1 × 10^8^ cells of hUCB-MSCs. Participants were followed for up to 5 years until study completion, death, withdrawal of consent, or loss to follow-up. During the follow-up period, patients received treatment according to their physician’s standard practice. Information about the use of DMARDs was collected.

This study was approved by the Institutional Review Board of Seoul Metropolitan Government–Seoul National University Boramae Medical Center (IRB No. 26-2016-44) and was conducted in accordance with the Good Clinical Practice guidelines and the Declaration of Helsinki. All participants provided written informed consent before participation.

### Safety profiles

The primary objective of this study was to evaluate the long-term safety of a single intravenous infusion of hUCB-MSCs over a 5-year follow-up period. Adverse events (AEs) and serious adverse events (SAEs) were recorded at 3, 6, and 12 months after infusion, and annually thereafter. SAEs were defined according to FDA criteria, including life-threatening events, required hospitalization, or resulting in significant disability, congenital anomaly, or death.^23^ AEs of special interest included serious infections, opportunistic infections,^24^ major adverse cardiovascular events (MACEs), thromboembolic events, and malignancies. Physical examinations, laboratory tests, and ECGs were performed at each visit.

AE causality was evaluated by the investigating physicians using a predefined clinical assessment framework. This assessment considered the timing between hUCB-MSC infusion and the event onset, biological plausibility, and alternative causes, including underlying disease activity and concomitant medications. All events were systematically reviewed based on these predefined clinical criteria to ensure a consistent and meaningful evaluation of causality. No independent adjudication committee was involved.

### Clinical assessments for disease activity

To assess the long-term safety profile in a clinical context, disease activity was tracked over time using the Disease Activity Score 28 (DAS28). These measures of clinical efficacy, along with changes in concomitant medications such as biologic or targeted synthetic DMARDs, were collected from medical records at baseline, week 4, months 3 and 6, and annually up to 5 years after infusion.

### Statistical analysis

Continuous variables were reported as mean ± standard deviation (SD), while categorical variables were presented as frequencies and percentages. Paired *t*-tests or Wilcoxon signed-rank tests were used to compare baseline and follow-up lab results, with Bonferroni correction applied for multiple comparisons. Statistical significance was set at *P* < 0.05.

## Results

### Patients

**Figure 1.**
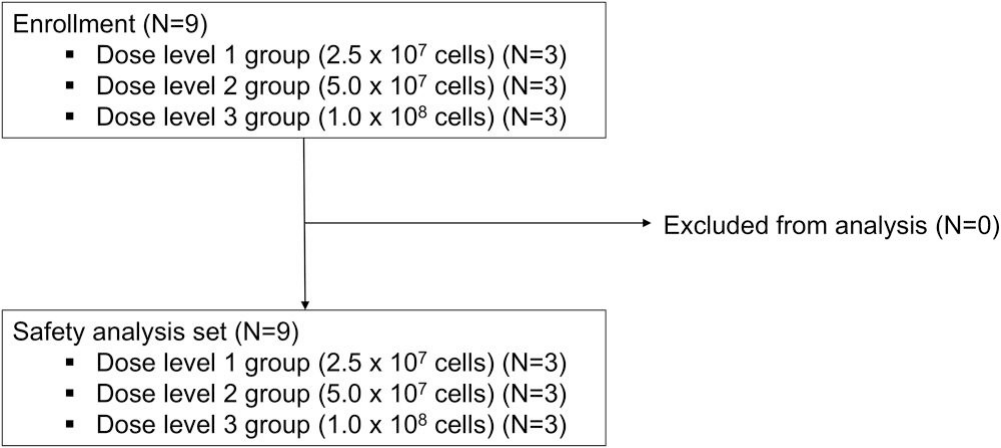
Patient selection process.

A total of 9 patients who participated in the CURE-iv trial were included in the long-term safety analysis and completed the 5-year study (Figure 1). Patients received a single intravenous infusion of 2.5 × 10^7^ (*n* = 3), 5.0 × 10^7^ (*n* = 3), and 1.0 × 10^8^ (*n* = 3) hUCB-MSCs, respectively. Demographics and baseline clinical characteristics of patients are summarized in Table 1. The mean ± SD age of the participants was 57.4 ± 10.0 years, and 77.8% were female. The mean ± SD disease duration of RA was 9.5 ± 8.7 years, with a baseline DAS 28-ESR of 4.53 ± 1.35 (Table 1). At baseline, all patients were prescribed MTX with a mean dose of 14.2 mg/week. Seven patients received systemic corticosteroids with a mean daily dose of 3.1 mg of prednisolone or its equivalent (Table 1). None had been previously prescribed biologic DMARDs.

**Table 1:** Baseline characteristics of the study population.

	All MSC doses (*n* = 9)
**Age, years**	57.4 ± 10.0
**Female**	7 (77.8)
**Disease duration, years**	9.5 ± 8.7
**BMI, kg/m^2^**	25.2 ± 0.9
**Swollen joint count**	2.4 ± 2.7
**Tender joint count**	11.8 ± 16.7
**Laboratory parameters**	
**Rheumatoid factor positivity**	6 (66.7)
**Anti-CCP positivity**	4 (44.4)
**ESR, mm/h**	23.3 ± 12.0
**CRP, mg/dL**	0.81 ± 1.12
**DAS28-ESR**	4.53 ± 1.35
**Pain VAS, mm**	64.8 ± 20.2
**HAQ**	0.69 ± 0.63
**Concomitant medication at baseline**	
**Methotrexate**	9 (100)
**Methotrexate dose, mg/week**	14.2 ± 0.9
**Oral corticosteroids use**	7 (77.8)
**Oral corticosteroid dose, mg/d^a^**	3.1 ± 0.8
**Prior bDMARD use**	0 (0)

Values are mean ± SD or *n* (%).

Abbreviations: bDMARD, biological disease modifying antirheumatic drug; BMI, body mass index; CCP, cyclic citrullinated peptide; CRP, C-reactive protein; DAS28, 28‐joint disease activity score; ESR, erythrocyte sedimentation rate; HAQ, health assessment questionnaire; MSC, mesenchymal stem cell; VAS, visual analog scale.

a Prednisolone or its equivalent.

### Summary of AEs

Over the 5-year study period, 97 AEs were observed across all patients (Table 2). The most common AEs were osteoarthritis (*n* = 4 [44.4%]) and nasopharyngitis (*n* = 4 [44.4%]), followed by abdominal pain or discomfort (*n* = 3 [33.3%]), osteoporosis or osteopenia (*n* = 3 [33.3%]), lumbar spinal stenosis (*n* = 2 [22.2%]), and hypertension (*n* = 2 [22.2%]). AEs such as diabetes mellitus, hypertriglyceridemia, peripheral edema, compression fracture, meniscus lesion, limb deformity, colon polyp, gastric ulcer, nausea, tinnitus, dizziness, insomnia, anemia, eosinophilia, and allergic rhinitis were reported in 1 patient each. Severe AEs occurred in 3 patients—1 in each dose group of hUCB-MSCs—but no life-threatening AEs or deaths were reported during the 5 years. A total of 9 SAEs were reported in 5 patients (55.6%) during the study (Table 2). The SAEs included cellulitis, compression fracture, limb deformity, lumbar spinal stenosis, colorectal polyp, diabetes mellitus, sinusitis, meniscus lesion, and arthralgia. None of these were considered related to the hUCB-MSCs infusion.

**Table 2:** Summary of adverse events up to 5 years.

	Total (*n* = 9)	Dose level 1 (*n* = 3)	Dose level 2 (*n* = 3)	Dose level 3 (*n* = 3)
**Any AE**				
**No. of patients (%)**	9 (100)	3 (100)	3 (100)	3 (100)
**No. of events**	97	37	30	30
**Severe AE**				
**No. of patients (%)**	3 (33.3)	1 (33.3)	1 (33.3)	1 (33.3)
**No. of events**	7	5	1	1
**Life-threatening AE**				
**No. of patients (%)**	0	0	0	0
**Any SAE**				
**No. of patients (%)**	5 (55.6)	3 (100)	1 (33.3)	1 (33.3)
**No. of events**	9	6	1	2
**Death**	0	0	0	0
**AEs reported in ≥1 of patients in any group, (%)**				
**Osteoarthritis**	4 (44.4)	0	1 (33.3)	3 (100)
**Nasopharyngitis**	4 (44.4)	2 (66.7)	0	2 (66.7)
**Abdominal pain or discomfort**	3 (33.3)	1 (33.3)	1 (33.3)	1 (33.3)
**Osteopenia/osteoporosis**	3 (33.3)	0	2 (66.7)	1 (33.3)
**Lumbar spinal stenosis**	2 (22.2)	2 (66.7)	0	0
**Hypertension**	2 (22.2)	1 (33.3)	1 (33.3)	0
**Diabetes mellitus**	1 (11.1)	1 (33.3)	0	0
**Hypertriglyceridemia**	1 (11.1)	1 (33.3)	0	0
**Peripheral edema**	1 (11.1)	1 (33.3)	0	0
**Compression fracture**	1 (11.1)	1 (33.3)	0	0
**Meniscus lesion**	1 (11.1)	0	0	1 (33.3)
**Limb deformity**	1 (11.1)	1 (33.3)	0	0
**Colon polyp**	1 (11.1)	1 (33.3)	0	0
**Gastric ulcer**	1 (11.1)	0	1 (33.3)	0
**Nausea**	1 (11.1)	1 (33.3)	0	0
**Tinnitus**	1 (11.1)	0	1 (33.3)	0
**Dizziness**	1 (11.1)	0	0	1 (33.3)
**Insomnia**	1 (11.1)	0	0	1 (33.3)
**Anemia**	1 (11.1)	0	1 (33.3)	0
**Eosinophilia**	1 (11.1)	0	1 (33.3)	0
**Allergic rhinitis**	1 (11.1)	0	0	1 (33.3)
**AEs of special interest**				
**Serious infections**	1 (11.1)	0	0	1 (33.3)
**Opportunistic infections**	0	0	0	0
**MACE**	1 (11.1)	0	0	1 (33.3)
**Thromboembolism**	0	0	0	0
**Malignancies**	0	0	0	0
**Benign tumor**				
**Benign ovarian tumor**	1 (11.1)	1 (33.3)	0	0
**Breast adenoma**	1 (11.1)	0	1 (33.3)	0

Dose levels of hUCB-MSCs: 1 = 2.5 × 10^7^ cells; 2 = 5.0 × 10^7^ cells; 3 = 1.0 × 10^8^ cells.

Abbreviations: AE, adverse event; MACE, major adverse cardiovascular event; No, number; SAE, serious adverse event.

### AEs of special interests

One case of serious infection (cellulitis) was observed in a patient who received a 1.0 × 10^8^ hUCB-MSCs infusion, which was resolved after antibiotic treatment (Table 2). One case of MACE, specifically heart failure, was also reported in a patient in the same dose group. The cellulitis event occurred more than 12 months after infusion in a patient receiving concomitant DMARD therapy, while the heart failure event occurred years later in a patient with pre-existing hypertension. Given the long interval between infusion and event onset, along with the presence of underlying risk factors, both events were considered unlikely to be related to the study intervention. Both events recovered without sequelae. Three years after the infusion, a benign ovarian tumor and a breast adenoma were reported in patients who received doses of 2.5 × 10^7^ and 5.0 × 10^7^ hUCB-MSCs, respectively. No cases of opportunistic infections, thromboembolism, or malignancy were reported during the 5-year follow-up period (Table 2).

Table 3 summarizes the distribution of long-term AEs stratified by the initial hUCB-MSC dose groups. While both events of special interest (cellulitis and heart failure) occurred in the highest-dose group, no consistent dose-dependent pattern in the frequency or severity of AEs was apparent in this small exploratory cohort.

**Table 3:** Distribution of long-term adverse events stratified by hUCB-MSC dose groups.

Adverse events	2.5 × 10^7^ cells (*n* = 3)	5.0 × 10^7^ cells (*n* = 3)	1.0 × 10^8^ cells (*n* = 3)
**Most common AEs, number of patients (events)**			
**Osteoarthritis**	0	1 (3)	3 (5)
**Nasopharyngitis/Common cold**	2 (5)	0	2 (2)
**Abdominal pain or discomfort**	1 (2)	1 (1)	1 (1)
**Osteoporosis or osteopenia**	0	2 (2)	1 (1)
**Lumbar spinal stenosis**	2 (2)	0	0
**Hypertension**	1 (2)	1 (1)	0
**AEs of special interest, number of patients**			
**Serious infection (Cellulitis)**	0	0	1
**Major adverse cardiovascular event (Heart failure)**	0	0	1
**Benign ovarian tumor**	1	0	0
**Breast adenoma**	0	1	0
**Malignancy**	0	0	0
**Opportunistic infection**	0	0	0
**Thromboembolism**	0	0	0

Data for common AEs presents the number of patients experiencing the event, with the total number of events indicated in parentheses. For AEs of special interest and SAEs, data presents the number of patients. AEs were mapped based on preferred terms from the clinical database.

Abbreviations: AEs, adverse events; hUCB-MSCs, human umbilical cord blood-derived mesenchymal stem cells.

### Occurrence of clinical AEs by organ systems over time

After 1 year of a single intravenous infusion of hUCB-MSCs, the incidence of AEs began to increase over time. Musculoskeletal AEs occurred consistently throughout the observation period, while infection and gastrointestinal-related AEs were observed 1 year after the administration of hUCB-MSCs (Figure S1).

### Laboratory parameters

Mean laboratory parameters remained stable over 5 years following the infusion of hUCB-MSCs (Figure S2). WBC count, along with absolute neutrophil and lymphocyte counts, hemoglobin, creatinine, total cholesterol, and liver function tests stayed within the normal range in all patients. One patient showed an increased triglyceride level 3 months after receiving hUCB-MSCs, which normalized without lipid-lowering therapy. Eosinophilia was observed in 1 patient 60 months after the infusion of hUCB-MSCs, without any associated skin rash or other clinical symptoms.

### Long-term disease activity and medication changes

**Figure 2.**
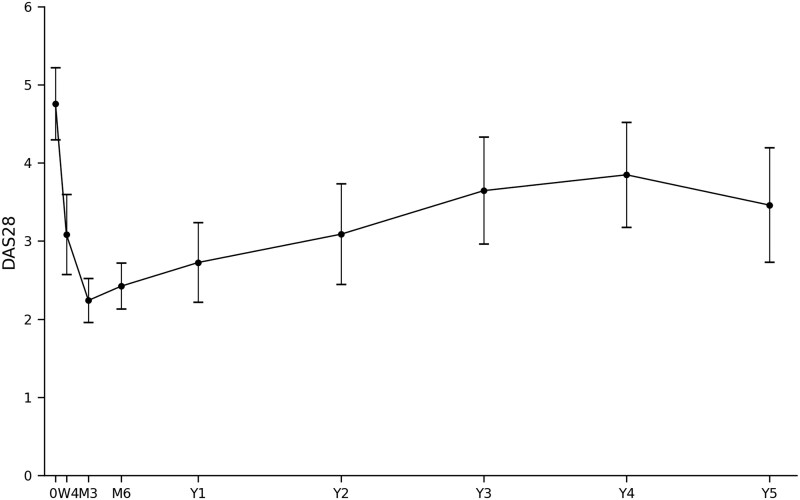
Long-term changes in disease activity over 5 years. Mean DAS28 values are shown from baseline to 5 years post-infusion (baseline, week 4, months 3 and 6, and annually thereafter). Error bars represent standard error of the mean. DAS28, Disease Activity Score 28; M, month; W, week; Y, year.

To provide clinical context for the long-term safety profile, we analyzed longitudinal changes in DAS28 and medications over 5 years. After a single intravenous infusion of hUCB-MSCs, disease activity decreased markedly, reaching its lowest levels between 3 and 6 months (mean ± SD DAS28 at baseline vs. 3 months: 4.76 ± 1.38 vs 2.24 ± 0.91). This clinical improvement was generally maintained up to 1 year after infusion (Figure 2). However, consistent with the chronic nature of RA and the single-dose approach, disease activity gradually returned toward baseline over the extended 5-year follow-up. During this period, 2 out of 9 patients (22.2%) required treatment escalation to biologic or targeted synthetic DMARDs (1 to tocilizumab and 1 to tofacitinib) to maintain disease control.

## Discussion

This study provides initial long-term safety data on hUCB-MSC therapy in patients with RA, with one of the longest follow-up periods currently available. Previous clinical studies have mainly concentrated on short-term or early-phase data, with long-term safety data remaining an unmet need in this field. Establishing the long-term safety of MSC-based therapies is especially important in RA, where prolonged use of immunomodulatory treatments has been linked to cumulative risks of severe infections, cardiovascular events, and malignancies.^4^^,^^10^^,^^25^ In this context, the present study provides additional observational safety data with a specific focus on monitoring these clinically relevant AEs.

Over the 5-year follow-up period, a single intravenous infusion of hUCB-MSCs was generally well tolerated. We prospectively monitored key AEs of clinical concern—including serious infections, MACE, thromboembolic events, and malignancies—and no such late-onset events were observed. One case of cellulitis requiring intravenous antibiotics and one case of heart failure were reported in the 1.0 × 10^8^ group; notably, both events resolved without sequelae and were considered unrelated to the study intervention. Additionally, no opportunistic infections or deaths occurred, and laboratory parameters remained stable throughout the 5-year follow-up. When considered within a risk-benefit framework, these safety findings are supported by the observed clinical efficacy. Disease activity showed a meaningful reduction after infusion, reaching its lowest levels at 3-6 months and remaining improved for up to 1 year. Although disease activity gradually returned toward baseline over time—consistent with the natural progression of RA following a single-dose treatment—the initial therapeutic effect indicates a favorable short-term benefit without evident long-term safety concerns. Overall, these findings offer a plausible risk-benefit assessment and provide initial reassurance regarding the long-term safety of hUCB-MSC therapy.

The immunomodulatory mechanisms of MSCs have been well-characterized in preclinical studies. MSCs promote an increase in Treg cells and interleukin-10 (IL-10)-expressing CD4+ T cells (Tr1), while suppressing T helper 1 (Th1) and Th17 pro-inflammatory subsets.^11^ They also modulate cytokine profiles by reducing pro-inflammatory cytokines, including TNF-α, IL-17, IL-4, and interferon-gamma, and enhancing anti-inflammatory cytokines such as IL-10 and transforming growth factor-beta.^11^^,^^26^ In addition, MSCs can polarize M1 macrophages toward an anti-inflammatory M2 phenotype^27^ and inhibit NF-B ligand (RANKL)-induced osteoclastogenesis, thereby reducing bone loss in collagen-induced arthritis models.^28^ Furthermore, MSCs have been shown to suppress B cell activation, reducing plasmablast differentiation and pro-inflammatory antibody production,^29^ and to modulate synovial fibroblast activity, leading to decreased production of IL-6, matrix metalloproteinases, and other inflammatory mediators.^30^ Preclinical animal studies consistently demonstrate that MSC-based therapies delay arthritis progression with favorable safety profiles across diverse tissue sources, MHC contexts, and routes of administration.^26^ Furthermore, the positive long-term safety profile may be partly due to the natural biological properties of hUCB-MSCs. Specifically, the low immunogenicity of hUCB-MSCs—marked by minimal expression of major histocompatibility complex class II and co-stimulatory molecules—likely helps prevent graft-versus-host reactions, severe infusion-related toxicities, and delayed alloimmune responses over the 5-year follow-up period.^31^^,^^32^ These biological properties offer a plausible mechanistic explanation for the observed safety profile. Several early-phase clinical studies have also explored MSC-based therapy in RA.^13–16^^,^^33^ In a phase 1 trial, a single intravenous infusion of autologous BM-MSCs (1.0 × 10^6^ cells) led to reductions in Th17 cells and IL-17A expression at 12 months, with no AEs.^14^ Allogeneic adipose-derived MSCs, administered intravenously at escalating doses (1.0 × 10^6^, 2.0 × 10^6^, or 4.0 × 10^6^ cells on days 1, 8, and 14), demonstrated an acceptable safety profile over 6 months, although 3 SAEs were reported, including 1 case of dose-limiting lacunar infarction, 1 case of peroneal nerve palsy, and 1 case of fever.^13^ Notably, infections were the most common AEs but were non-serious and no opportunistic infections were observed.^13^ In another study, human UC-MSCs (4 × 10^7^ cells), combined with DMARDs, provided clinical improvements and reduced inflammation over 3 years, with a favorable safety profile.^33^

While previous studies have shown promising evidence for the safety and potential effectiveness of MSC-based therapies in RA, most have focused on shorter follow-up periods, varied MSC sources, preparation methods, and dosing regimens, and limited systematic evaluation of important long-term safety outcomes. The current study provides 5-year safety data on hUCB-MSC therapy, including a prospective assessment of clinically relevant AEs such as severe infections, opportunistic infections, which are particularly concerning in RA management. Additionally, the use of a well-characterized and standardized hUCB-MSC product with advantageous source properties, including noninvasive collection, higher proliferative capacity, and lower immunogenicity,^20^^,^^21^ distinguishes this study from earlier trials using more diverse MSC preparations. These findings further support their potential as a well-tolerated and clinically feasible option in RA.

In chronic autoimmune diseases like RA, long-term safety should be viewed within a risk-benefit framework. In this context, our parallel analysis of disease activity offers valuable clinical insight into safety outcomes. Disease activity decreased markedly after infusion, reaching its lowest point within the first year, demonstrating the short-term therapeutic effect of hUCB-MSCs. Over the subsequent years, disease activity gradually moved back toward baseline during the 5-year follow-up, which may reflect the progressive course of RA and the temporary nature of a single-dose treatment.^1^^,^^11^ Notably, despite this gradual decline in efficacy, a small number of patients required escalation to targeted synthetic or biologic DMARDs, and no long-lasting immune-related safety issues were observed. These results indicate a favorable short-term risk–benefit profile, though maintaining long-term disease control might require repeated treatments.

This study has several limitations. First, as a follow-up to a phase I trial, this study involved a small cohort (*n* = 9) and was not statistically powered to detect meaningful safety differences across dose levels. Specifically, the distribution of AEs across the 3 dose groups is provided solely for descriptive transparency and should not be interpreted as a formal dose-response analysis given the small number of participants per cohort. Although events of special interest, such as cellulitis and heart failure, were observed in the highest-dose group, no definitive dose-dependent trends or causal links can be established from these subgroup observations, and rare or very late AEs cannot be excluded. Second, the open-label, single-arm design without a placebo or active control group prevents definitive causal conclusions about long-term safety. Without a comparative control, it remains difficult to distinguish potential hUCB-MSC-related AEs from the expected background incidence of such events in RA populations with similar age and treatment exposure. Finally, while our efficacy data suggest the need for repeated administrations, this study assessed only the safety of a single intravenous infusion. Although our findings indicate an acceptable safety profile after a single dose, repeated infusions could change the long-term risk profile. Therefore, future large-scale, randomized controlled phase II/III trials with multi-dose regimens and long-term follow-up are needed.

In conclusion, this 5-year follow-up study suggests that a single intravenous infusion of hUCB-MSCs has an acceptable long-term safety profile in patients with RA. While the small sample size and absence of a control group necessitate cautious interpretation, the scarcity of 5-year longitudinal safety data for hUCB-MSCs in RA makes these findings a valuable preliminary clinical reference. These results justify further clinical research with larger, controlled studies to assess the feasibility of multi-dose regimens.

## Supplementary Material

szag062_Supplementary_Data

## Data Availability

The data supporting the findings of this study are available from the corresponding author upon reasonable request.
