# Supplementary material for: Long-term safety of intravenous human umbilical cord blood-derived mesenchymal stem cell therapy in rheumatoid arthritis: a 5-year follow-up study
Source: Stem Cells Transl Med. 2026 Aug 5;15(8):szag062. doi: 10.1093/stcltm/szag062 (PMC13441169; doi:10.1093/stcltm/szag062)
Supplement: Supplementary file 1 [file szag062_supplementary_data.docx]

**Supplementary Information**

**Long-term safety of intravenous human umbilical cord blood-derived mesenchymal stem cell therapy in rheumatoid arthritis: a 5-year follow-up study**

Eun Hye Park^1,2^, Min Jung Kim^3^, Kyung-sun Kang^4,5^, Kichul Shin^3,6^

^1^Division of Rheumatology, Department of Internal Medicine, Chung-Ang University College of Medicine, Seoul, Republic of Korea

^2^Division of Rheumatology, Department of Internal Medicine, Chung-Ang University Gwangmyeong Hospital, Gwangmyeong, Republic of Korea

^3^Division of Rheumatology, Department of Internal Medicine, Seoul Metropolitan Government–Seoul National University Boramae Medical Center, Seoul, Republic of Korea

^4^Institute for Stem Cell Regenerative Medicine, Kangstem Biotech, Seoul, Republic of Korea

^5^Adult Stem Cell Research Center, College of Veterinary Medicine, Seoul National University, Seoul, Republic of Korea

^6^Department of Internal Medicine, College of Medicine, Seoul National University, Seoul, Republic of Korea

**Corresponding author**:

Kichul Shin, M.D., Ph.D.

Seoul Metropolitan Government–Seoul National University Boramae Medical Center

20 Boramae-ro-5-gil, Dongjak-gu, Seoul, 07061, Republic of Korea

Tel: +82 2870 3204, Fax: +82 2870 3866

Email: [kideb1@snu.ac.kr](mailto:kideb1@snu.ac.kr)

**Supplementary Figure Legends**

**Supplementary Figure 1. Clinical adverse events by organ system up to 5 years**

**Supplementary Figure 2. Longitudinal changes in laboratory parameters over 5 years.**

(a) WBC count, (b) absolute neutrophil count, (c) absolute lymphocyte count, (d) eosinophil count, (e) hemoglobin, (f) creatinine, (g) aspartate aminotransferase, (h) alanine aminotransferase, and (i) total cholesterol, and (j) triglyceride. Error bars represent standard error of the mean.
AST, aspartate aminotransferase; ALT, alanine aminotransferase.
